# Treatment expectations of patients and clinicians: a cross-sectional study

**DOI:** 10.3389/fpsyt.2024.1447405

**Published:** 2024-08-22

**Authors:** Jiarui Li, Chunfeng Xiao, Tao Li, Yanping Duan, Yinan Jiang, Lili Shi, Xia Hong, Wenqi Geng, Jiaojiao Hu, Yufei Wang, Bindong Dai, Jinya Cao, Jing Wei

**Affiliations:** ^1^ Department of Psychological Medicine, Peking Union Medical College Hospital, Chinese Academy of Medical Sciences & Peking Union Medical College, Beijing, China; ^2^ 4^+^4 Medical Doctor Program, Chinese Academy of Medical Sciences & Peking Union Medical College, Beijing, China

**Keywords:** patients, clinicians, doctors, treatment expectation, personalized medicine, patient safety, doctor-patient relationship, treatment outcome

## Abstract

**Importance:**

Understanding treatment expectations of patients and their clinicians is of great importance in improving personalized medical services and enhancing patient safety systems.

**Objective:**

To investigate treatment expectations of patients and their clinicians and compare differences between both, by using a pair of validated structured assessment tools covering three key aspects/dimensions of clinical interests.

**Design, setting, and participants:**

This single-center cross-sectional study was conducted at Peking Union Medical College Hospital in China. The study enrolled patients aged 16 years and older receiving inpatient care and their clinicians. Patient recruitment was conducted from March 2023 to November 2023.

**Assessments:**

In addition to demographic and clinical characteristics, this study employed two validated structured assessment tools to evaluate treatment expectations among patients and their clinicians: the Hospitalized Patients’ Expectations for Treatment Scale-Patient version (HOPE-P) and its counterpart, the Hospitalized Patients’ Expectations for Treatment Scale-Clinician version (HOPE-C).

**Results:**

A total of 233 patients (mean [SD] age, 52.3 [15.1] years; 108 [46.4%] female) along with their clinicians, who numbered 75 in total were enrolled in this study. The distribution of total scores for HOPE-P and HOPE-C displayed similar patterns, with most scores concentrated in the higher range (above 50% of the full score). The mean HOPE-P total score was higher than that of HOPE-C (mean [SD] score, 38.78 [4.86] vs 37.49 [4.32]; *t* = 3.12, *P* = 0.002). In Dimension 2, the HOPE-P score was higher than HOPE-C (23.67 [3.20] vs 21.72 [3.03]; *t* = 6.98, *P* < 0.001). However, in Dimensions 1 and 3, HOPE-P scored lower than HOPE-C (13.37 [2.44] vs 13.84 [1.73]; *t* = -2.384, *P* < 0.018; 1.74 [1.14] vs 1.94 [1.00]; *t* = -2.00, *P* = 0.047). Certain demographic and clinical characteristics led to variations in patients’ treatment expectations, including marital status, monthly family income, and smoking history.

**Conclusions and relevance:**

This cross-sectional study revealed significant differences between patients’ and doctors’ treatment expectations. Notably, it highlighted the need for clinicians to focus on rationalizing patients’ expectations concerning treatment outcomes.

**Trial Registration Chinese Clinical Trial Registry Identifier:**

ChiCTR2300075262.

## 1 Introduction

Existing research has already focused on observing and understanding the potential impact of patients’ treatment expectations on health outcomes (1, 2). Although the number of relevant studies is limited, they have yielded some enlightening results that are valuable for clinical practice. In addition to being a pivotal mechanism in the placebo and nocebo effects, where subjective and physiological changes are induced by inert or non-specific treatment components, mounting evidence underscores the significant role of patient expectations in influencing treatment outcomes across a variety of medical conditions (3–9).

Furthermore, transitioning the treatment process toward patient-centered, personalized medical services requires the integration of patients’ treatment expectations and satisfaction (10). Additionally, overlooking patients’ treatment expectations may impact patient safety. On one hand, the level of patients’ treatment expectations can influence their adherence to the treatment (11, 12). On the other hand, patients’ treatment expectations can affect doctors’ medical decisions, potentially leading to inappropriate prescriptions (13). Both factors may directly or indirectly affect patient safety. Hence, a deeper understanding of patients’ and clinicians’ treatment expectations is crucial for improving patient health outcomes, enhancing personalized medical services, and bolstering patient safety systems.

However, previous research concerning treatment expectations of patients and clinicians exhibits considerable heterogeneity, and this has been identified as a major limitation in several systematic reviews and meta-analyses (9, 14). This includes variations in focus, with some studies concentrating on probability expectations (what patients think will happen) and others on value expectations (what patients would like to happen) (15). Additionally, even when the focus is consistent, the specific dimensions/aspects and research methodologies used to understand treatment expectations of patients and clinicians vary. This diversity hinders the ability to synthesize previous findings and draw solid conclusion. Moreover, few studies have compared the treatment expectations of patients and clinicians in the same dimensions/aspects (14, 16, 17). Therefore, research employing a comprehensive set of structured assessment tools to understand and explore the differences in treatment expectations of patients and clinicians in dimensions/aspects of clinical interests is urgently needed.

Our previous studies have developed and validated a set of structured assessment tools for assessing the treatment expectations of patients and clinicians (18, 19). These tools share an identical dimensional structure and scoring system, ensuring consistency in the assessment process. By using these validated tools, we can reduce the variability in measurement methods across studies, facilitating more reliable comparisons and synthesis of findings. This standardization allows for a clearer understanding of treatment expectations and contributes to reducing heterogeneity in the field. In this cross-sectional study, we will apply these tools to understand the differences in treatment expectations between patients and their clinicians. This will provide important evidence-based support for further improving personalized medical services and enhancing patient safety systems.

## 2 Methods

### 2.1 Trial design

Following institutional review board approval from the ethics committee of Peking Union Medical College Hospital. This cross-sectional study was conducted at Peking Union Medical College Hospital in China. All participants provided their electronic informed consent, and additional parental informed consent was obtained for children younger than 18 years. This study followed the Strengthening the Reporting of Observational Studies in Epidemiology (STROBE) Statement: guidelines for reporting observational studies. Patients and the public were not involved in the design, or conduct, or reporting, or dissemination plans of this research.

### 2.2 Participants

The study enrolled participants receiving inpatient care, and their clinicians (defined as a medical doctor responsible for the treatment and care of the patients enrolled in the study) at Peking Union Medical College Hospital, regardless of wards. Clinical departments were selected by computerized random sampling. During the study period, patients who met the inclusion criteria were recruited voluntarily from the relevant departments. Inclusion criteria were: (1) age 16 years or older; (2) had been receiving inpatient care for more than 24 h. Exclusion criteria were: (1) not having fluent Chinese language skills; (2) cognitive impairment, severe hearing or visual impairment or intellectual disability preventing adherence to the study procedure. Participants recruitment was conducted from March 2023 to November 2023.

### 2.3 Assessments

In addition to demographic and clinical characteristics, this study employed two validated structured assessment tools to evaluate treatment expectations among patients and their clinicians: the Hospitalized Patients’ Expectations for Treatment Scale-Patient version (HOPE-P) and its counterpart, the Hospitalized Patients’ Expectations for Treatment Scale-Clinician version (HOPE-C) (18, 19) (**Appendix**). Both tools share an identical dimensional structure (three dimensions: doctor-patient communication expectation [items 1 to 3], treatment outcome expectation [items 4–8], disease management expectancy [item 9]) and scoring system (Likert five-point, with each item ranging from 1 to 5, full score=45), facilitating direct comparison of assessment outcomes. These are self-rated instruments designed to be universally applicable across various diseases. Heterogeneity in disease conditions was handled by ensuring that the assessment tools were generic and not disease-specific. Our previous work has already confirmed the validity and reliability of these tools when applied to inpatients and their clinicians in general hospital settings in China.

### 2.4 Sample size calculation

In this study, a formal sample size calculation was not conducted, a decision influenced by the study’s exploratory nature and the practical constraints of data availability. Lack of a formal sample size calculation may limit the confidence in our findings. Nevertheless, our primary goal was to generate hypotheses and identify trends rather than to test specific hypotheses.

### 2.5 Statistical analysis

Continuous variables are presented as mean (SD) or median (IQR). Categorical variables are presented as numbers and percentages. We used independent-sample *t* test, or one-way analysis of variance (ANOVA) to compare the differences in HOPE-P and HOPE-C scores across various dimensions and in total scores among different variables. An independent-sample *t* test was used rather than a paired *t* test because the study aimed to compare the overall differences in treatment expectations between patients and clinicians, not the matched expectations for individual patient-clinician pairs. Kernel Density Estimation (KDE) was applied to visualize the distribution of HOPE-P and HOPE-C scores, providing a smoothed estimate of the probability density function (20). Correlations between the items, dimensions and total scores of HOPE-P and HOPE-C and variables were calculated using the Spearman correlation coefficient. All the statistical analyses were performed with R programming language (version R 4.2.1, https://www.R-project.org). Data were analyzed in December 2023.

## 3 Results

### 3.1 Participants

Between March 2023 and November 2023, of the 260 patients initially screened for participation, 27 were excluded based on the exclusion criteria: 17 due to cognitive impairment, and 10 due to severe hearing or visual impairment. Therefore, this cross-sectional study enrolled a total of 233 patients along with their clinicians, who numbered 75 in total (**Table 1**). It is important to note that some clinicians were responsible for multiple patients within the study. Among 233 patients (mean [SD] age, 52.3 [15.1] years; 108 [46.4%] female) from seven different wards, most were receiving inpatient care in surgical wards, with 90 (38.6%) in Urology, 45 (19.3%) in General Surgery, and 36 (15.5%) in Orthopedics.

**Table 1:** Demographic and clinical characteristics of the sample and distribution of HOPE-P and HOPE-C scores.

Characteristic	Participants, No. (%)	HOPE-P				HOPE-C			
		D1, mean (SD)	D2, mean (SD)	D3, mean (SD)	Total, mean (SD)	D1, mean (SD)	D2, mean (SD)	D3, mean (SD)	Total, mean (SD)
Sex									
Female	108 (46.4)	13.3 (2.39)	23.52 (3.39)	1.72 (1.1)	38.54 (4.95)	13.96 (1.45)	21.6 (3.03)	1.87 (1.01)	37.44 (3.93)
Male	125 (53.6)	13.44 (2.49)	23.79 (3.03)	1.76 (1.18)	38.99 (4.79)	13.74 (1.94)	21.82 (3.04)	1.99 (0.99)	37.54 (4.66)
Age, years									
≤50	90 (38.6)	13.14 (2.43)	23.22 (3.45)	1.87 (1.13)	38.23 (5.16)	14.01 (1.55)	22.34 (2.67)	1.86 (1.00)	38.21 (3.71)
>50	143 (61.4)	13.52 (2.45)	23.94 (3.01)	1.66 (1.14)	39.13 (4.66)	13.73 (1.83)	21.32 (3.18)^*^	1.99 (0.99)	37.04 (4.63)^*^
Ethnicity									
Han	227 (97.4)	13.4 (2.43)	23.71 (3.18)	1.75 (1.15)	38.86 (4.81)	13.82 (1.74)	21.69 (3.04)	1.94 (0.99)	37.46 (4.34)
Other	6 (2.6)	12.5 (3.02)	21.83 (3.60)	1.33 (0.52)	35.67 (6.19)	14.5 (1.22)	22.67 (2.58)	1.67 (1.21)	38.83 (3.76)
Education Level									
Elementary	16 (6.9)	14.06 (1.53)	24.13 (1.93)	1.63 (1.26)	39.81 (2.64)	14.13 (1.02)	20.88 (3.48)	1.88 (1.09)	36.88 (4.53)
Junior	50 (21.5)	12.96 (3.03)	23.4 (3.75)	1.7 (1.05)	38.06 (5.9)	13.74 (1.86)	21.66 (3.09)	1.9 (1.05)	37.3 (4.23)
High School	42 (18)	13.19 (2.71)	23.64 (2.88)	1.74 (1.21)	38.57 (5.14)	13.71 (1.86)	21.38 (3.22)	1.95 (0.94)	37.05 (4.53)
College or Higher	125 (53.6)	13.51 (2.16)	23.72 (3.22)	1.78 (1.15)	39.01 (4.53)	13.89 (1.71)	21.96 (2.88)	1.95 (0.99)	37.8 (4.3)
Residence									
Urban	193 (82.8)	13.37 (2.47)	23.72 (3.32)	1.75 (1.15)	38.84 (4.97)	13.81 (1.74)	21.76 (3.01)	1.95 (0.99)	37.52 (4.37)
Rural	40 (17.2)	13.38 (2.35)	23.43 (2.58)	1.7 (1.14)	38.5 (4.39)	13.98 (1.69)	21.53 (3.15)	1.88 (1.02)	37.38 (4.17)
Marital Status									
Married	190 (81.5)	13.42 (2.49)	23.77 (3.26)	1.72 (1.18)	38.91 (4.94)	13.85 (1.77)	21.61 (3.16)	1.9 (0.96)	38.18 (3.64)
Single	28 (12.0)	13.21 (1.64)	23.54 (2.22)	1.96 (0.96)	38.71 (2.97)	13.89 (1.57)	22.11 (2.45)	2.18 (1.22)	37.35 (4.51)
Divorced	5 (2.1)	13.6 (1.14)	23.4 (2.07)	2 (1.22)	39 (3.81)	14.4 (1.34)	22.4 (2.07)	1.4 (0.89)	38.2 (2.39)
Widowed	5 (2.1)	15 (0)	25 (0)	1 (0)	41 (0)	12.8 (1.3)	21.4 (2.07)	2.6 (0.89)	36.8 (3.63)
Other	5 (2.1)	10.8 (4.55)	19.2 (5.07)	2 (0.71)	32 (8.92)	13.8 (1.79)	23.4 (1.82)	1.8 (1.1)	39 (2.55)
Health Insurance									
No	11 (4.7)	13.82 (1.99)	23.82 (2.44)	1.55 (1.21)	39.18 (4.35)	14 (2.41)	21.91 (3.33)	1.45 (0.82)	37.36 (4.67)
Yes	222 (95.3)	13.35 (2.46)	23.66 (3.24)	1.75 (1.14)	38.76 (4.9)	13.83 (1.7)	21.71 (3.02)	1.96 (1)	37.5 (4.32)
Employment Status									
Employed	78 (33.5)	13.6 (1.78)	23.77 (2.52)	1.74 (1.12)	39.12 (3.39)	13.9 (2.06)	22.15 (3.28)	1.85 (0.97)	37.9 (4.92)
Unemployed	155 (66.5)	13.26 (2.72)	23.61 (3.5)	1.74 (1.16)	38.61 (5.46)	13.81 (1.55)	21.5 (2.88)	1.98 (1.01)	37.29 (4)
Monthly Family Income									
<4,000 CNY	48 (20.6)	12.71 (3.16)	22.69 (4.17)	1.81 (1.25)	37.21 (6.31)	13.83 (1.88)	21.58 (3.29)	2.02 (1.08)	37.44 (4.64)
4,000-8,000 CNY	86 (36.9)	13.78 (2.33)	24.14 (2.74)^*^	1.76 (1.21)	39.67 (4.46)^*^	13.76 (1.91)	21.63 (3.22)	1.85 (0.98)	37.23 (4.78)
>8000 CNY	99 (42.5)	13.34 (2.07)	23.73 (2.95)	1.7 (1.03)	38.77 (4.21)	13.92 (1.49)	21.86 (2.73)	1.97 (0.97)	37.75 (3.75)
Living Arrangement									
Living Alone	15 (6.4)	13.2 (1.78)	23.07 (2.46)	1.87 (0.83)	38.13 (3.29)	13.73 (1.44)	22.4 (2.13)	2 (1.07)	38.13 (2.75)
Livingwith Others	218 (93.6)	13.39 (2.48)	23.71 (3.24)	1.73 (1.16)	38.83 (4.96)	13.85 (1.75)	21.67 (3.08)	1.93 (0.99)	37.45 (4.42)
Alcohol History									
No	153 (65.7)	13.52 (2.24)	23.77 (3.07)	1.67 (1.06)	38.95 (4.57)	13.71 (1.8)	21.47 (3.03)	1.91 (0.97)	37.09 (4.45)
Yes	80 (34.3)	13.1 (2.78)	23.46 (3.44)	1.89 (1.28)	38.45 (5.4)	14.09 (1.58)	22.19 (2.98)	1.99 (1.05)	38.26 (3.98)
Smoking History									
No	157 (67.4)	13.56 (2.08)	23.93 (2.56)	1.61 (1.02)	39.1 (3.96)	13.9 (1.51)	21.87 (2.8)	1.94 (1.02)	37.7 (3.89)
Yes	76 (32.6)	12.99 (3.04)	23.12 (4.2)	2.01 (1.33)*	38.12 (6.31)	13.72 (2.13)	21.41 (3.45)	1.93 (0.96)	37.07 (5.12)
Exercise Frequency in Winter (Weekly)									
No	79 (33.9)	13.41 (2.43)	23.62 (3.13)	1.75 (1.21)	38.77 (4.69)	13.78 (1.71)	21.19 (3.02)	1.97 (1.05)	36.95 (4.27)
Less Than1 Hours	59 (25.3)	13.1 (2.73)	23.32 (3.33)	1.83 (1)	38.25 (5.34)	13.81 (2.06)	22.17 (3.29)	1.85 (0.98)	37.83 (5.08)
1-2 Hours	52 (22.3)	13.25 (2.33)	23.69 (3.2)	1.83 (1.23)	38.77 (4.87)	13.83 (1.71)	22.13 (3.04)	2.1 (1.14)	38.06 (4.33)
More Than2 Hours	43 (18.5)	13.84 (2.18)	24.19 (3.18)	1.51 (1.08)	39.53 (4.56)	14 (1.27)	21.56 (2.55)	1.79 (0.67)	37.35 (3.17)
Exercise Frequency in Summer (Weekly)									
No	72 (30.9)	13.33 (2.49)	23.54 (3.21)	1.72 (1.19)	38.6 (4.9)	13.93 (1.38)	21.35 (2.75)	1.86 (0.95)	37.14 (3.77)
Less Than1 Hours	56 (24.0)	13.48 (2.3)	23.66 (3.23)	1.73 (1.02)	38.88 (4.66)	13.57 (2.4)	21.77 (3.81)	2.05 (1.05)	37.39 (5.85)
1-2 Hours	58 (24.9)	13.05 (2.47)	23.86 (2.24)	1.95 (1.22)	38.86 (4.29)	13.91 (1.67)	22.24 (2.89)	1.97 (1.12)	38.12 (4.12)
More Than2 Hours	47 (20.2)	13.7 (2.53)	23.62 (4.12)	1.53 (1.1)	38.85 (5.79)	13.94 (1.33)	21.57 (2.49)	1.87 (0.82)	37.38 (3.12)
Department									
Internal	62 (26.6)	13.11 (2.61)	23.23 (3.58)	1.68 (1.07)	38.02 (5.32)	14.18 (1.87)	20.74 (3.71)	1.45 (0.88)	36.37 (5.01)
Pulmonology	2 (0.9)	15 (0)	25 (0)	1 (0)	41 (0)	12.5 (2.12)	20.5 (0.71)	3 (1.41)	36 (0)
Immunology	22 (9.4)	11.77 (3.39)	22.59 (4.8)	1.59 (1.01)	35.95 (7.14)	14.14 (1.36)	18.23 (2.39)	1.36 (0.58)	33.73 (3.1)
Endocrinology	23 (9.9)	13.78 (1.7)	23.61 (2.35)	1.78 (1.17)	39.17 (3.31)	14.65 (0.83)	21.91 (2.52)^bb^	1.65 (1.15)	38.22 (2.81)^b^
Cardiology	15 (6.4)	13.8 (1.82)	23.33 (3.37)	1.73 (1.1)	38.87 (4.34)	13.73 (3.17)	22.67 (5.04)^bb^	1.07 (0.26)^c^	37.47 (8.03)
Surgery	171 (73.4)	13.47 (2.38)	23.82 (3.04)	1.77 (1.17)	39.06 (4.67)	13.72 (1.67)	22.07 (2.66)^*^	2.11 (0.98)^*^	37.9 (3.99)^*^
Orthopedics	36 (15.5)	12.5 (2.37)	23.08 (3.89)	2.56 (1.42)	38.14 (5.64)	14.17 (2.14)^d^	23.03 (3.41)^ddd^	1.92 (1.16)	39.11 (4.96)^dd^
General Surgery	45 (19.3)	13.09 (2.35)	23.56 (2.68)	1.78 (1)	38.42 (4.37)	14.38 (1.34)^ddd^	23.44 (2.19)^ddd^	2.07 (1.07)^aa^	39.89 (3.14)^ddd^
Urology	90 (38.6)	14.04 (2.25)	24.26 (2.78)	1.44 (0.98)	39.74 (4.33)	13.21 (1.43)^aa^	21 (2.02)	2.21 (0.84)^aaa^	36.42 (3.32)

D1~Dimension 1 (doctor– patient communication expectation); D2~Dimension 2 (treatment outcome expectation); D3~Dimension 3 (disease management expectancy).

Student’s t-test or one-way ANOVA with Scheffé’s post-hoc test (for three or more groups) was used as appropriate. Compared with the first characteristic: ^*^ *P* < 0.05; Compared with Orthopedics: ^a^ *P* <0.05, ^aa^ *P* <0.01, ^aaa^ *P* < 0.001; Compared with Immunology: ^b^ *P* < 0.05, ^bb^ *P* ^<^0.01; Compared with Pulmonology: ^c^ *P* <0.05; Compared with Urology: ^d^ *P* < 0.05, ^dd^ *P* < 0.01, ^ddd^ *P* < 0.001.

In terms of monthly family income, patients were categorized into three groups: less than 4000 CNY (less than approximately 600 USD), 4000-8000 CNY (approximately 600-1200 USD), and more than 8000 CNY (more than approximately 1200 USD).

### 3.2 Variations in treatment expectations: patients vs clinicians

**Figure 1:**
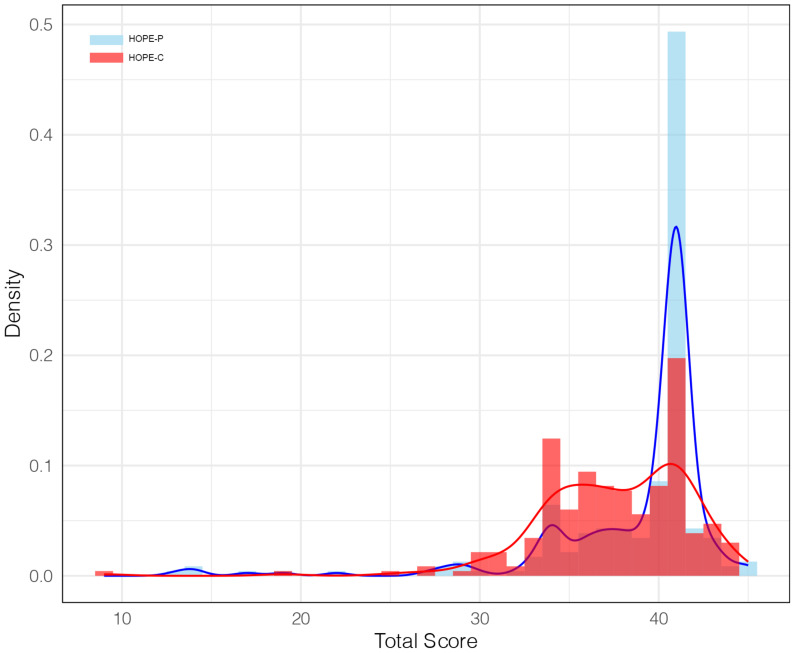
Distribution of HOPE-P and HOPE-C total scores. This figure presents the probability density histograms with kernel density estimations (KDE) for HOPE-P (in blue) and HOPE-C (in red) total scores. The x-axis represents the total score values, while the y-axis represents the density, with the area under each curve summing to one. The KDE curves provide a smoothed estimate of the score distributions, indicating the likelihood of score occurrence within the population.

The distribution of total scores for HOPE-P and HOPE-C displayed similar patterns, with most scores concentrated in the higher range (above 50% of the full score) (**Figure 1**). Notably, most HOPE-P scores were clustered at 41 points (115 patients [49.4%]), while the distribution of HOPE-C scores in the higher range was relatively more dispersed.

The scores across different dimensions of the HOPE-P and HOPE-C revealed distinct patterns. The majority of patients achieved full points in Dimensions 1 and 2, with 124 patients (53.2%) and 173 patients (74.2%) respectively. However, in Dimension 3, the most common score was the lowest possible, with 143 patients (61.4%) receiving a score of 1. In contrast, HOPE-C scores were concentrated in Dimension 1, with the majority of clinicians (130 [55.8%]) scoring the maximum of 15 points. Scores were more dispersed in Dimensions 2 and 3, with the highest score of 25 points achieved by 59 clinicians (25.3%) and 20 points by 56 clinicians (24.0%) in Dimension 2, while in Dimension 3, the most common scores were 1 and 2 points (both were 92 [39.5%]).

**Figure 2:**
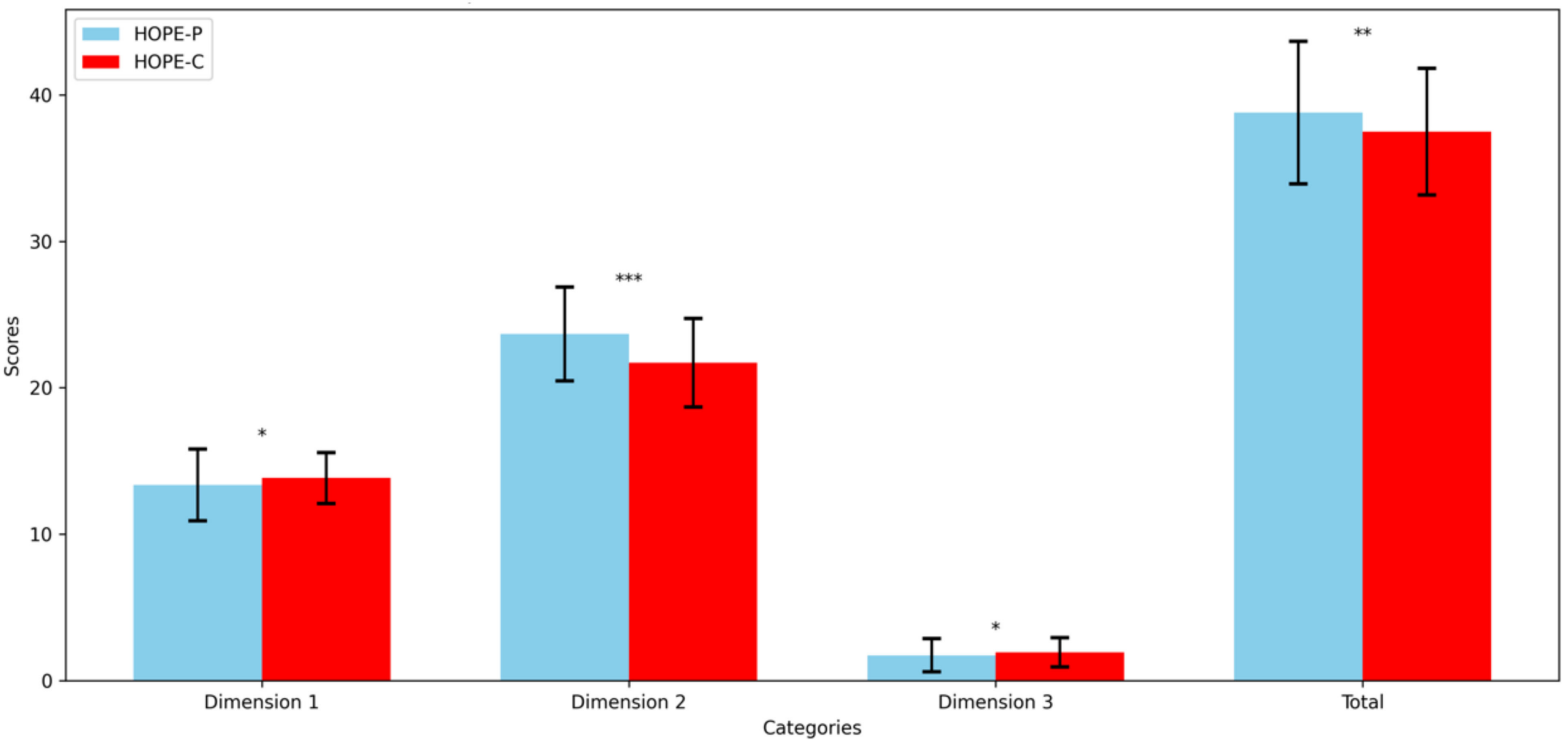
Paired comparison of HOPE-P and HOPE-C scores across dimensions and total score. This bar chart illustrates the mean scores for HOPE-P (blue bars) and HOPE-C (red bars) across three dimensions and the overall total score. Error bars represent one standard deviation from the mean, indicating the variability within each group. Statistically significant differences between the scores, as determined by student’s *t* test, are indicated by asterisks directly above the corresponding boxplots, with * denoting *P* < 0.05, ** denoting *P* < 0.01, and *** denoting *P* < 0.001. These annotations highlight dimensions where perceptions of patients and their clinicians significantly diverge.

There were significant differences between HOPE-P and HOPE-C scores across dimensions and total scores (**Figure 2**). The mean HOPE-P total score was higher than that of HOPE-C (mean [SD] score, 38.78 [4.86] vs 37.49 [4.32]; *t* = 3.12, *P* = 0.002). In Dimension 2, the HOPE-P score was higher than HOPE-C (23.67 [3.20] vs 21.72 [3.03]; *t* = 6.98, *P* < 0.001). However, in Dimensions 1 and 3, HOPE-P scored lower than HOPE-C (13.37 [2.44] vs 13.84 [1.73]; *t* = -2.384, *P* = 0.018; 1.74 [1.14] vs 1.94 [1.00]; *t* = -2.00, *P* = 0.047).

Further detailed comparisons of scoring variations across specific items reveal that, except for items 2 and 3, significant differences were observed in the scores of HOPE-P and HOPE-C across other items (**Supplementary 1**). Particularly in item 6 (“Through this hospitalization, the disease can be cured”), a more pronounced visible difference in score distribution between HOPE-P and HOPE-C was noted, with HOPE-P scores predominantly clustered at the full score of 5 points.

### 3.3 Variations in treatment expectations by demographic and clinical characteristics

Analysis of the distribution of HOPE-P and HOPE-C scores across dimensions and total scores showed no significant differences based on sex, ethnicity, education level, residence, employment status, living arrangement, alcohol history, exercise frequency in summer/winter, and health insurance coverage (**Table 1**).

Patients over 50 years old showed significantly lower scores in Dimension 2 and the total score of HOPE-C compared to those 50 years old or younger, as evaluated by their clinicians (21.32 [3.18] vs. 22.34 [2.67], *t* = 2.541, *P* = 0.012; 37.04 [4.63] vs. 38.21 [3.71], *t* = 2.022, *P* = 0.044). Married patients scored significantly higher in Dimension 2 and the total score compared to those with other marital statuses (23.77 [3.26] vs. 19.2 [5.07], *P* = 0.039; 38.91 [4.94] vs. 32.00 [8.92], *P* = 0.041). Patients with a monthly income of 4000–8000 reported significantly higher scores in Dimension 2 and the total score compared to those earning less than 4000 (24.14 [2.74] vs 22.69 [4.17], *P* = 0.041; 39.67 [4.46] vs 37.21 [6.31], *P* = 0.018). Patients with a smoking history had higher scores in Dimension 3 of HOPE-P compared to non-smokers (2.01 [1.33] vs 1.61 [1.02], *P* = 0.012).

Compared to patients in internal medicine, those in surgical departments had significantly higher scores in Dimensions 2 and 3, and the total score of HOPE-C, as assessed by their clinicians (22.07 [2.66] vs 20.74 [3.71], *t* = -3.011, *P* = 0.003; 2.11 [0.98] vs 1.45 [0.88], *t* = -4.663, *P* < 0.001; 37.90 [3.99] vs 36.37 [5.01], *t* = -2.410, *P* = 0.017).

There were significant differences in Dimension 1 scores of HOPE-P among patients in various internal wards (F_[3,58]_ = 3.515, *P* = 0.021), although *post-hoc* analysis did not reveal differences between pairs of groups. In surgical wards, significant differences were observed in Dimensions 1 and 3 scores of HOPE-P (F_[2,168]_ = 6.598, *P* = 0.002; F_[2,168]_ = 13.278, *P* < 0.001). Patients in urology scored significantly higher in Dimension 1 of HOPE-P compared to those in orthopedics (14.04 [2.25] vs 12.5 [2.37], *P* = 0.004). Orthopedic patients had significantly higher scores in Dimension 3 of HOPE-P than those in general surgery and urology (2.56 [1.42] vs 1.78 [1.00], *P* = 0.007; 2.56 [1.42] vs 1.44 [0.98], *P* < 0.001).

Significant differences were observed in Dimensions 2 and 3, and the total score of HOPE-C among patients from different internal wards (F_[3,58]_ = 7.109, *P* < 0.001; F_[3,58]_ = 3.997, *P* = 0.012; F_[3,58]_ = 3.783, *P* = 0.015). Clinicians in endocrinology and cardiology scored significantly higher in Dimension 2 of HOPE-C compared to immunology (21.91 [2.52] vs 18.23 [2.39], *P* = 0.005; 22.67 [5.04] vs 18.23 [2.39], *P* = 0.002). For Dimension 3, clinicians in pulmonology scored significantly higher than those in cardiology (3.00 [1.41] vs 1.07 [0.26], *P* = 0.028). Clinicians in endocrinology scored significantly higher in the total score of HOPE-C compared to those in immunology (38.22 [2.81] vs 33.73 [3.10], *P* = 0.023).

There were significant differences in Dimensions 1 and 2, and the total score of HOPE-C among clinicians in various surgical wards (F_[2,168]_ = 9.931, *P* < 0.001; F_[2,168]_ = 18.909, *P* < 0.001; F_[2,168]_ = 15.759, *P* < 0.001). Clinicians in orthopedics and general surgery scored significantly higher in Dimension 1 of HOPE-C than those in urology (14.17 [2.14] vs 13.21 [1.43], *P* = 0.011; 14.38 [1.34] vs 13.21 [1.43], *P* < 0.001). They also scored higher in Dimension 2 (23.03 [3.41] vs 21.00 [2.02], *P* < 0.001; 23.44 [2.19] vs 21.00 [2.02], *P* < 0.001) and in the total score of HOPE-C (39.11 [4.96] vs 36.42 [3.32], *P* = 0.001; 39.89 [3.14] vs 36.42 [3.32], *P* < 0.001).

### 3.4 Correlation of treatment expectations between patients and clinicians

There was no significant correlation between most of the items, and the dimensions and total scores of HOPE-P and HOPE-C (**Table 2**).

**Table 2:** Correlations between HOPE-P and HOPE-C scores across items, dimensions and total score.

Item No.	Correlation	*P*
1	-0.030	0.651
2	-0.155	0.018
3	-0.138	0.035
4	-0.102	0.120
5	-0.032	0.624
6	0.008	0.906
7	-0.029	0.661
8	-0.060	0.360
9	0.020	0.757
Dimension 1	-0.112	0.089
Dimension 2	-0.043	0.509
Dimension 3	0.020	0.757
Total Score	-0.017	0.800

## 4 Discussion

This cross-sectional study employed validated and comparable structured assessment tools, providing evidence to understand the current levels of treatment expectations among patients and their clinicians, and offering evidence-based foundations for comprehending the differences in treatment expectations between these groups.

Patients and clinicians often have differing treatment expectations, especially regarding treatment outcomes. A study conducted by Karpinski et al. on patients with partial- or full-thickness tears of the rotator cuff scheduled for arthroscopic repair found that the expectations of patients regarding their surgery differed from the surgeon’s assessment. While patients prioritized regaining range of motion, surgeons emphasized the importance of pain relief (21). Furthermore, there was a significant difference between patients and clinicians in their perception of the degree of treatment benefits, with patients having higher early treatment expectations. Mancuso et al. found that the concordance between patients’ and surgeons’ expectations was moderate, primarily due to patients expecting complete improvement, whereas surgeons anticipated a range of outcomes, from a lot to a little improvement (22). Notably, surgeons’ expectations aligned more closely with patient-reported fulfillment of expectations two years after the surgery compared to the initial expectations of patients.

In this study, both patients and doctors exhibited high levels of treatment expectations, yet these expectations differed significantly. Patients overall had significantly higher treatment expectations than clinicians. Specifically, patients had higher expectations for treatment outcomes than clinicians, but their expectations for doctor-patient communication and disease management were lower. These results suggest that their treatment expectations were more aligned with realistic and immediate benefits and also reveal differences in treatment expectations partly due to the gap in medical information between patients and clinicians. Patients’ higher expectations may be driven by a lack of comprehensive medical knowledge, leading them to anticipate more immediate and favorable outcomes. Their expectations are often shaped by anecdotal information from peers or media, which may not always align with medical realities. In contrast, clinicians’ expectations are grounded in clinical evidence and experience, focusing on realistic outcomes based on the patient’s condition and treatment possibilities. It also indicates that, although clinicians have gradually become more aware and genuinely attentive to patients’ needs for doctor-patient communication, there is still a need to focus more on understanding patients’ treatment outcome expectations. This suggests that clinicians need to discuss different expectations with patients early in the treatment process, particularly in reaching a consensus on treatment outcomes.

In light of the significance of shared decision-making and patient empowerment within the doctor-patient relationship, our findings underscore the necessity for clinicians to transparently communicate treatment prognoses to patients (23, 24). Such clarity not only fosters a shared understanding between patients and clinicians but also empowers patients, ensuring their voices are respected in the decision-making process. This approach aligns with the principles of personalized medicine, where treatment is tailored to the individual’s specific conditions and expectations, enhancing the patient safety system. This is also crucial for increasing patient adherence to treatment and overall satisfaction, thereby facilitating the realization of patient-centered personalized medicine and the further enhancement of patient safety systems (11, 25). Our study suggests that clinicians particularly need to work toward rationalizing patients’ expectations regarding the curability of diseases through treatment.

Patients’ treatment expectations can be effectively shaped through various strategies. Verbal affirmations of treatment benefits have been shown to modulate expectations positively (26, 27). Additionally, employing an empathetic interaction style can influence patient attitudes, and discussing patients’ beliefs and concepts about treatment also plays a significant role (28, 29). Recent clinical intervention studies have demonstrated that brief psychological interventions can optimize patients’ expectations, leading to improved health outcomes (30, 31). In light of our study results, it is particularly important to facilitate the exchange of medical information between patients and clinicians, aiming to balance their treatment expectations and minimize the existing gaps in medical knowledge.

Certain potential demographic and clinical characteristics led to variations in patients’ treatment expectations, including marital status, monthly family income, and smoking history. Patients who are married, compared to those with other marital statuses such as being separated, tend to have higher treatment outcome expectations and overall treatment expectations, possibly due to the motivation to continue fulfilling family responsibilities. Patients with a monthly income of 4000–8000, as opposed to those earning less than 4000, exhibit higher treatment outcome expectations, suggesting that economic status may influence treatment expectations, with middle-income patients having higher expectations or demands for treatment compared to their lower-income counterparts. Smokers, compared to non-smokers, have lower disease management expectancy. However, receiving inpatient care in internal or surgical departments does not affect patients’ treatment expectations. Clinicians tend to have lower treatment outcome expectations and overall treatment expectations for older patients. This aligns with the clinical reality where clinicians often base their prognostic expectations on a patient’s age. Additionally, surgeons typically have higher treatment outcome expectations and overall expectations, but lower disease management expectancy, compared to physicians. This suggests that clinicians in internal medicine generally adopt a more conservative attitude to patients’ treatment benefits and prognosis. Due to the limitations of the sample size in this study, interpretations of treatment expectations based on more detailed departmental classifications may not be entirely solid. In the future, more research is warranted on identifying factors influencing treatment expectations of patients and clinicians.

Interestingly, we found that there are no correlations between treatment expectations among patients and their clinicians. This may reveal that patients and clinicians each hold their own distinct treatment expectations, which are often misaligned. Such differences should be given due attention and carefully managed, given their impact on the advancement of personalized medicine and patient safety.

### 4.1 Limitations

This study has several limitations that warrant consideration. First, the limited number of relevant studies and constraints in the research environment have resulted in a restricted sample size for our study, potentially limiting the generalizability of our findings. Second, the study population was drawn from a single hospital in China. While the results provide valuable insights into treatment expectations within this specific context, caution is warranted when generalizing these findings to other populations. Cultural, demographic, and geographic differences can significantly influence treatment expectations. For example, treatment expectations in Western countries may differ due to variations in healthcare systems, patient-clinician communication styles, and cultural attitudes toward healthcare. Therefore, future studies should include more diverse demographic and geographic contexts to validate and expand upon these results. Third, the study was conducted primarily in surgical wards, which may limit the generalizability of the findings. Patients in surgical wards may have different treatment expectations compared to those in non-surgical or chronic care settings. Future studies should encompass a broader range of clinical settings, such as internal medicine, pediatrics, and chronic disease management, to enhance the applicability of the findings and provide a more comprehensive understanding of treatment expectations across various medical disciplines. Fourth, any conclusions drawn from this study should be treated with caution since the sample comes from a single center. Deficits in patient-clinician communication can vary widely between clinicians, and specific centers may have unique policies to address these issues. These factors may influence the results and limit their generalizability. Future multicenter studies are necessary to confirm these findings and account for variability across different healthcare settings. Fifth, the heterogeneity in disease conditions and individual patient characteristics may influence treatment expectations. Patients with different diseases and varying severities of conditions might have diverse expectations. This variability underscores the need for future studies to consider stratified analyses based on specific diseases or conditions to provide more precise insights. Lastly, the number of patients and clinicians differed, with each clinician caring for multiple patients. This distribution could potentially influence the results, particularly if a single clinician had a large proportion of patients under their care. This concentration might skew the findings and limit their generalizability. It is important to note that genuine attentiveness to patients’ needs and clinician-patient communication is now the standard of care in many health systems. However, caution should be exercised when making generalizing statements based on this single study, as practices may vary significantly across different healthcare environments. Therefore, the results should be interpreted in the context of the specific healthcare setting of this study and future research should aim to validate these findings in diverse healthcare environments. Moreover, to enhance the generalizability of the findings, future research should aim to include diverse study populations and settings. This approach will help to ensure that the results are applicable across different demographic groups and healthcare environments, thus providing a more comprehensive understanding of treatment expectations in various contexts.

## 5 Conclusion

Differences exist between patients’ and doctors’ treatment expectations, especially regarding treatment outcome expectations. Multicenter studies with large samples, are needed to confirm these findings and to identify the factors influencing the treatment expectations of patients and/or clinicians. Such studies would further guide the development of personalized medicine and the improvement of patient safety systems.

## Data Availability

The raw data supporting the conclusions of this article will be made available by the authors, without undue reservation.
