# Supplementary material for: Treatment expectations of patients and clinicians: a cross-sectional study
Source: Front Psychiatry. 2024 Aug 22;15:1447405. doi: 10.3389/fpsyt.2024.1447405 (PMC11374636; doi:10.3389/fpsyt.2024.1447405)
Supplement: Supplementary file 1 [file DataSheet1.pdf]

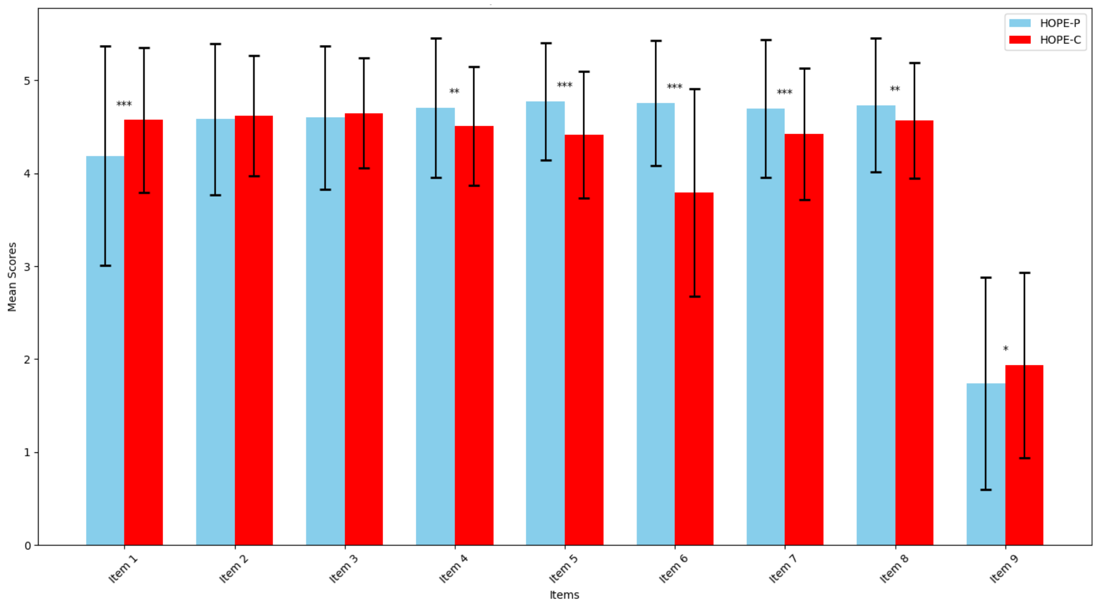

eFigure 1. Item-wise Comparison of HOPE-P and HOPE-C Scores

This bar chart provides a comparative analysis of the mean scores for each item of the HOPE-P (in sky blue) and HOPE-C (in red) instruments. The error bars represent the standard deviation, indicating the spread of the scores. Statistically significant differences between the HOPE-P and HOPE-C scores, as determined by student’s t-tests, are denoted above the bars: * for $P < 0.05$, ** for $P < 0.01$, and *** for $P < 0.001$.
