# Supplementary material for: Treatment expectations of patients and clinicians: a cross-sectional study
Source: Front Psychiatry. 2024 Aug 22;15:1447405. doi: 10.3389/fpsyt.2024.1447405 (PMC11374636; doi:10.3389/fpsyt.2024.1447405)
Supplement: Supplementary file 2 [file DataSheet2.pdf]

Hospitalized Patients' Expectations for Treatment Scale - Patient Version (HOPE-P)

Instruction: Please assess the importance of the following items according to your expectation towards this hospitalization.

Q1. The doctor listens to my opinions on treatment
A. Least important
B. Less important
C. Of average importance
D. Fairly important
E. Very important

Q2. During this hospitalization, the doctor fully explains the state of illness to me and negotiates medical decisions with me
A. Least important
B. Less important
C. Of average importance
D. Fairly important
E. Very important

Q3. During this hospitalization, the doctor is caring
A. Least important
B. Less important
C. Of average importance
D. Fairly important
E. Very important

Q4. Through this hospitalization, the disease can be definitely diagnosed
A. Least important
B. Less important
C. Of average importance
D. Fairly important
E. Very important

Q5. Through this hospitalization, symptoms can be improved
A. Least important
B. Less important
C. Of average importance
D. Fairly important
E. Very important

Q6. Through this hospitalization, the disease can be cured

A. Least important
B. Less important
C. Of average importance
D. Fairly important
E. Very important

Q7. Through this hospitalization, I can restore work/family functions
A. Least important
B. Less important
C. Of average importance
D. Fairly important
E. Very important

Q8. Through this hospitalization, I can take care of myself
A. Least important
B. Less important
C. Of average importance
D. Fairly important
E. Very important

Q9. After this hospitalization, I need to maintain long-term treatment
A. Least important
B. Less important
C. Of average importance
D. Fairly important
E. Very important

The HOPE-P was designed as a self-administered expectation measurement tool, designated generally for hospitalized patients of all specialties and indexed as nine items and three domains describing what patients expect from hospitalization. Two domains evaluated patient expectations, including expectations of doctor–patient communication (items 1–3) and treatment outcomes (items 4–8), with higher scores indicating higher expectations from doctors and hospitalization. One domain (item 9) evaluated patients' cognition of disease management, which is a more realistic expectancy of the disease, and backward scoring was used for this item, with higher scores indicating higher expectations that future long-term treatment will not be needed after hospitalization. Each item is rated on a five-point Likert scale ranging from 1 to 5.

Hospitalized Patients' Expectations for Treatment Scale – Clinician Version (HOPE-C)

Instruction: Based on your expectations for this patient's current hospitalization and treatment, please evaluate the feasibility of the following items as you perceive it.

Q1. Listening to the patient's treatment opinions
A. Completely unfeasible
B. Largely unfeasible
C. Uncertain
D. Possibly feasible
E. Completely feasible

Q2. Informing the patient and making joint medical decisions
A. Completely unfeasible
B. Largely unfeasible
C. Uncertain
D. Possibly feasible
E. Completely feasible

Q3. Demonstrating compassion to the patient during treatment
A. Completely unfeasible
B. Largely unfeasible
C. Uncertain
D. Possibly feasible
E. Completely feasible

Q4. Achieving a definitive diagnosis during hospitalization
A. Completely unfeasible
B. Largely unfeasible
C. Uncertain
D. Possibly feasible
E. Completely feasible

Q5. Improvement of symptoms through treatment
A. Completely unfeasible
B. Largely unfeasible
C. Uncertain
D. Possibly feasible
E. Completely feasible

Q6. Complete cure of disease through treatment
A. Completely unfeasible

B. Largely unfeasible
C. Uncertain
D. Possibly feasible
E. Completely feasible

Q7. Restoration of work/family functionality post-treatment
A. Completely unfeasible
B. Largely unfeasible
C. Uncertain
D. Possibly feasible
E. Completely feasible

Q8. Patient’s ability for self-care post-treatment
A. Completely unfeasible
B. Largely unfeasible
C. Uncertain
D. Possibly feasible
E. Completely feasible

Q9. Need for long-term maintenance treatment post-hospitalization
A. Completely unfeasible
B. Largely unfeasible
C. Uncertain
D. Possibly feasible
E. Completely feasible

The HOPE-C is a self-administered tool for measuring treatment expectations, intended for use by clinicians across various specialties. It comprises nine items, organized into three distinct domains. Two domains evaluated clinicians’ expectations, including expectations of doctor–patient communication (items 1–3) and treatment outcomes (items 4–8), with higher scores indicating higher expectations. One domain (item 9) evaluated clinicians’ cognition of disease management, which is a more realistic expectancy of the disease, and backward scoring was used for this item, with higher scores indicating higher expectations that future long-term treatment will not be needed after hospitalization. Each item is rated on a five-point Likert scale ranging from 1 to 5.
